# Occurrence of multiple genotype infection caused by *Leishmania infantum* in naturally infected dogs

**DOI:** 10.1371/journal.pntd.0007986

**Published:** 2020-07-27

**Authors:** Elisa Cupolillo, Amanda S. Cavalcanti, Gabriel Eduardo Melim Ferreira, Mariana Côrtes Boité, Fernanda Nazaré Morgado, Renato Porrozzi

**Affiliations:** Laboratório de Pesquisa em Leishmaniose, Instituto Oswaldo Cruz, Fiocruz, Rio de Janeiro, RJ, Brasil; Institut de Recherche pour le Développement, FRANCE

## Abstract

Genetic polymorphisms in natural *Leishmania* populations have been reported in endemic areas. Microsatellite typing is a useful tool to elucidate the genetic variability of parasite strains, due to its capability for high-resolution mapping of genomic targets. The present study employed multilocus microsatellite typing (MLMT) to explore the genotypic composition of *Leishmania infantum* in naturally infected dogs by genotyping parasites infecting different tissues with or without *in vitro* expansion. Eighty-six samples were collected from 46 animals in an endemic region of visceral leishmaniasis (VL). MLMT was performed for 38 spleen samples and 48 *L*. *infantum* cultures isolated from different tissues. Of the 86 samples, 23 were effectively genotyped by MLMT, identifying nine multilocus genotypes (MLG; referred to as MLG A–I). MLGs A, B and C were detected in more than one type of tissue and in more than one sample. Conversely, MLG D-I were uniquely detected in one sample each. The results showed that multiple genotype infections occur within a single host and tissue. Paired sample analysis revealed the presence of different MLMT alleles in 14 dogs, while the same MLG allele was present in 15 animals. STRUCTURE analysis demonstrated the presence of two populations; 13 samples displayed a similar admixture of both ancestral populations, and these were not assigned to any population. Only samples for which Q ≥ 0.70 after CLUMPP alignment were considered to be part of Population 1 (POP1) or Population 2 (POP2). POP2 comprised the majority of samples (n = 54) compared to POP1 (n = 19). This study presents evidence of multiple genotype infections (caused by *L*. *infantum*) in dogs in an area with high VL transmission. Further investigations must be undertaken to determine the effects of multiple infection on the host immune response and disease dynamics and treatment.

## Author summary

American visceral leishmaniasis (VL) is a parasitic disease caused by the protozoan *Leishmania infantum*. This parasite can infect humans and animals and is transmitted by sand flies. Domestic dogs are considered an important host, and like humans, they can manifest the disease or present asymptomatic infections. Studies have identified genetic variations among *L*. *infantum* parasites from different endemic regions in the American continent. For other parasitic diseases (e.g., malaria), studies have suggested that multigenetic infection predicts the development of symptoms and can lead to a high level of transmission. However, the effects of the genetic composition of *Leishmania* parasites on VL need to be ascertained. This study used highly variable microsatellite markers to investigate multigenotype *L*. *infantum* populations among naturally infected dogs living in an area in which VL is highly prevalent. Samples obtained from different tissues were examined to identify the occurrence of multiple genotypes in the same animal and even within the same tissue.

## Introduction

*The Leishmania donovani* complex comprises two major species: *Leishmania infantum* (syn. *Leishmania chagasi*) and *Leishmania donovani*. These parasites cause visceral leishmaniasis (VL) around the world. *L*. *infantum* is associated with a zoonotic epidemiological cycle, while *L*. *donovani* is mostly associated with an anthroponotic cycle. In the zoonotic cycle observed in the Americas, the Middle East, Central Asia, China and the Mediterranean, dogs represent the most important domestic reservoir host and display clinical characteristics similar to those seen in humans with the disease [1].

In the Old World, both human and canine VL are associated with several *L*. *infantum* zymodemes [2]. Conversely, in the Americas, IOC/Z1 = MON1 is highly predominant. Using high-resolution genomic targets (such as microsatellites), which are useful in exploring variability among closely related strains, it is possible to identify different genotypes within the same zymodeme [3, 4]. Microsatellite-based analyses have revealed different multilocus genotypes (MLGs) circulating in the Americas [5] and have detected at least three different genetic populations (i.e., Population 1 [POP1], Population 2 [POP2] and Population 3 [POP3]) in Brazil [6]. POP1 is widely distributed in most Brazilian endemic regions. POP2 is well dispersed but predominant in the state of Mato Grosso. POP3 is less well dispersed and mainly comprises strains found in Mato Grosso do Sul. Several genotypes have been revealed to be infecting dogs in these regions [6].

Parasite infections are often characterized by a mix of distinct genotypes [7]. Such multiple genotype infections are likely more difficult for the immune defense of a host to control; thus, the probability that an infection will thrive is greater [8]. Distinct genotypes have been described that share the same milieu within a host, and such coexistence can either promote competition or cooperation among genotypes [8–10]. The genetic composition of the coinfecting pathogen population can significantly affect infection outcomes [11]. For instance, in malaria patients, studies have suggested that multiclonal infection predicts the risk of subsequent clinical malaria [12, 13] and that a high transmission intensity is maintained by a high level of mixed infection [14].

In canine visceral leishmaniasis (CVL), the infection outcome is a consequence of the interactions between the parasite and the host genetic background [15]; however, the direct effect of protozoan genotypes on disease dynamics remains unclear. Despite the relevance of multiple genotype infections, genetic characterization of *Leishmania* has rarely been attempted [16, 17], especially by using naturally infected animal and human tissues without *in vivo* or *in vitro* expansion.

Recently, whole genome sequencing (WGS) was employed as a proof-of-principle for the sequencing of *L*. *donovani* genomes directly from clinical samples of patients with VL [18]. Pairwise comparisons between the parasite genomes in clinical samples and the derived *in vitro*-cultured promastigotes showed genomic differences suggesting the presence of polyclonal infections in patients. However, the authors noted that direct analysis of the *Leishmania* genome in clinical samples is still hampered by the presence of high levels of human DNA and the large variation in the parasite load. Furthermore, this approach needs to be tested on samples from other hosts, including animal reservoirs and insect vectors, and in different host tissues. While many drawbacks still limit the use of WGS, microsatellite analysis represents an alternative for analyzing *Leishmania* parasites that infect different hosts without the potential bias caused by parasite culture, which may vary the average genomic content along *in vitro* maintenance [19]. The *Leishmania* genome is relatively rich in microsatellites [20], which represent important molecular markers to assess variability within species and between closely related strains. Thus, targeting microsatellites is a promising option for direct tissue studies [16]. Herein, we hypothesized that *Leishmania*-infected dogs from areas highly endemic for human VL are prone to polyclonal infection. We employed microsatellites to type DNA derived from spleen samples of dogs presenting different clinical scores and variable parasite loads. Furthermore, we typed DNA isolated from spleen and other tissues obtained from cultured parasites. Microsatellites are locus-specific and highly polymorphic. The results obtained herein portray polyclonal infections caused by *Leishmania* parasites in vertebrate hosts and depict differences between clinical samples and derived culture isolates.

## Methods

### Animals, sample collection, *Leishmania* parasite isolation, culture and species identification

**Fig 1:**
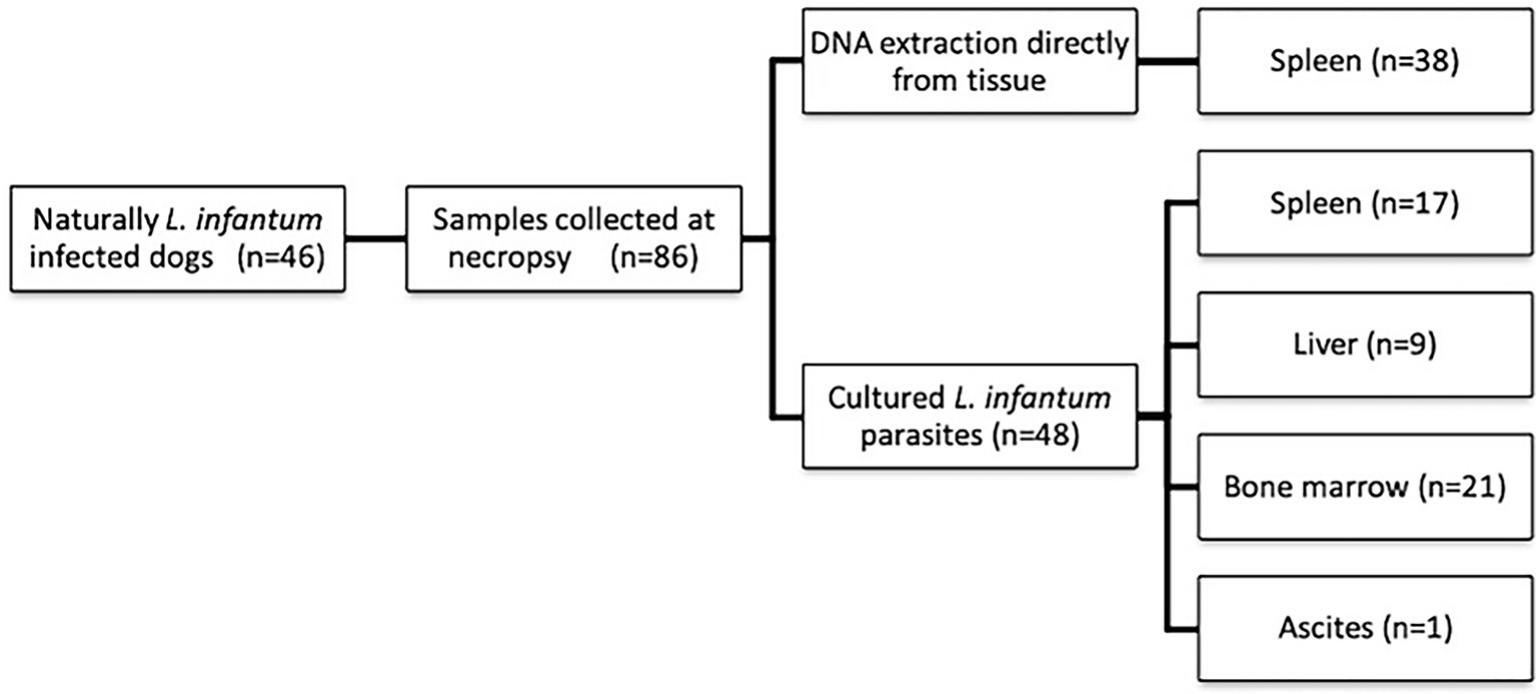
Flowchart outlining the sample collection. DNA was directly obtained from tissues, body fluid or *Leishmania infantum* parasites isolated and maintained in culture media. All samples were subjected to MLMT analysis. The number of tissues or Leishmania cultures analyzed is shown in parentheses.

This work was conducted with convenience samples. Samples were collected from 46 *Leishmania*-seropositive dogs destined to be euthanized (with the informed consent of the dogs’ owners) at the Center for Zoonosis Control in four endemic municipalities (i.e., Rondonópolis, Barra do Garças, Várzea Grande and Cuiabá) in the state of Mato Grosso, Brazil. These animals were euthanized, as they tested positive for *Leishmania* infection based on the rapid test (Dual Path Platform—DPP CVL, BioManguinhos, FIOCRUZ) and ELISA, according to the Brazilian Ministry of Health recommendations. The clearance of the Fundação Oswaldo Cruz Animal Ethics Committee was not required, as the samples were obtained *postmortem*, and the animals were not interfered for the purposes of this project. Fig 1 and S1 Table show all the samples included in this study in relation to the animal identification, the tissue collected and the type of sample (i.e., the tissue or parasite isolated in the culture) used for the molecular analysis. Information on clinical signs and parasite load in the spleen was gathered from our previous study [21].

Immediately after euthanasia, spleen and liver fragments, bone marrow aspirate and ascites were harvested for parasite isolation or DNA extraction. *Leishmania* parasites were isolated by maintaining spleen and liver fragments, bone marrow and ascites aspirates in culture conditions. The samples were seeded in a biphasic blood agar base and NNN-Schneider-Drosophila medium (Sigma-Aldrich, St. Louis, MO, USA) supplemented with 10% (v/v) heat-inactivated fetal bovine serum (FBS) (Sigma-Aldrich), 1.8% (v/v) penicillin, streptomycin and amphotericin (Sigma-Aldrich) and maintained at 25°C. Positive and non-contaminated cultures were maintained in the previously mentioned biphasic medium until the number of parasites required to perform the first *in vitro* passage was reached. Parasites from passages 2–5 were grown in liquid medium in 25 cm^3^ flasks for 3 days and then harvested for DNA extraction.

*Leishmania* species identification was performed by multilocus enzyme electrophoresis for parasites successfully isolated and maintained in culture or by PCR-RFLP of the *hsp*70 gene, following well-established protocols [22, 23].

To obtain DNA directly from the tissues of positive animals, only spleen tissues were employed and were stored in a buffer solution (10 mM NaCl, 10 mM EDTA, and 10 mM Tris HCl) at -20°C.

### DNA extraction from tissues and culture of parasites

Promastigotes from positive cultures were expanded at 25°C in Schneider’s insect medium, pH 7.2, supplemented with 10% (v/v) FBS and 2% (v/v) male human urine. The parasites were grown to a density of 1×10^9^ cells/mL (late log phase) and washed twice with PBS (pH 7.2) before DNA extraction.

Total DNA was extracted from spleen and from *L*. *infantum* promastigotes kept *in vitro*. DNA extraction was carried out using the Wizard Genomic DNA Purification System (Promega, Madison, WI, USA) following the manufacturer's instructions, which included a prior digestion phase with 17.5 μL of proteinase K (20 mg/mL) for 12 h at 55°C. The DNA was dissolved in 100 μL of tris EDTA buffer (TE buffer).

### PCR amplification and microsatellite markers

Fourteen primers conjugated with fluorophores HEX or 6FAM were used for microsatellite amplification (as previously described in [24]). PCR was performed in a volume of 25.0 μL containing 1X reaction buffer, 1.5 mM MgCl_2_, 2.5 mM deoxyribonucleotide triphosphate (dNTPs), 2U Taq polymerase (GoTaq, DNA Polymerase; Promega) and 100 ng DNA. Amplification conditions were achieved using Veriti equipment (Applied Biosystems, Foster City, CA/US) and consisted of an initial denaturation step at 95°C for 5 minutes, followed by 35 cycles at 95°C for 30 seconds, an annealing temperature for 30 seconds (S2 Table) and 72°C for 1 minute and a final extension at 72°C for 10 minutes.

Each amplicon (1.0 μL) was mixed with formamide (Hi-Di Formamide, Life Technologies) and a ladder marker (GeneScan 500 ROX Size Standard, Life Technologies). Fragment-length screening was performed with 500 ROX (Applied Biosystems) as the size standards using an ABI3130XL Genetic Analyzer (Applied Biosystems). The length analyses were performed on the Peak Scanner using Software v1.0 (available at http://www.appliedbiosystems.com). The results were standardized based on size profiles from the *L*. *infantum* reference strain IOC/L0579 (MHOM/BR/1974/PP75).

### Data analysis

A multilocus genotype (MLG) was assigned only to samples presenting the complete 14 microsatellite marker profile. Differences in at least one marker were enough to assign samples to a specific MLG. Samples with one or more missing data were not assigned to any MLG.

Population genetics algorithms were used to describe the clustering characteristics of the MLGs based on a previous study (see [6]). STRUCTURE software (v2.3.4) was used to infer the population structure. Next, Bayesian statistics and Markov Chain Monte Carlo simulations were used to estimate the assigned proportion of each individual belonging to each population (membership coefficient Q). The data for each value of K (from 1 to 15) were submitted on the STRUCTURE Harvester software v0.6.1 (available at http://taylor0.biology.ucla.edu/structureHarvester/, accessed on July 2019) for the estimation of delta K and the preparation of the indfiles for the software CLUMPP (v1.1.2), which was used to perform alignments from the Q values of each K STRUCTURE population. The resulting bar plots were visualized using Microsoft Excel software, and individuals were assigned to the cluster for which they exhibited the highest Q value. Finally, the Microsatellite Analyzer software was used to determine the fixation index (F_ST_) with significance (p) tested with 1,000 permutations.

## Results and discussion

Overall, 86 samples collected from 46 animals were analyzed (Fig 1). MLMT was performed by employing DNA derived directly from 38 spleen samples from L. infantum-infected dogs and from 48 *L*. *infantum* cultures isolated from different tissues. For 29 dogs, more than one sample type (tissue or parasite culture) was analyzed. For five animals, MLMT was performed only on *Leishmania* cultured isolates from different tissues (S1 Table).

Of the 14 loci analyzed, nine were polymorphic: Li45–24, Li41–56, Lm2TG, Lm4TA, Li 71–70, Li 71–52, Klist 70–39, Li 71–33 and Li 22–35. Alleles for each sample and marker are presented in S1 Table.

All samples were typed as *L*. *infantum* before the MLMT analyses. DNA from tissues was subjected to *Leishmania* species identification by PCR-RFLP *hsp*70, and cultured parasites were identified by isoenzyme electrophoresis in accordance with the protocols adopted by the *Leishmania* Collection (http://clioc.fiocruz.br/) from Fiocruz [22, 23].

**Fig 2:**
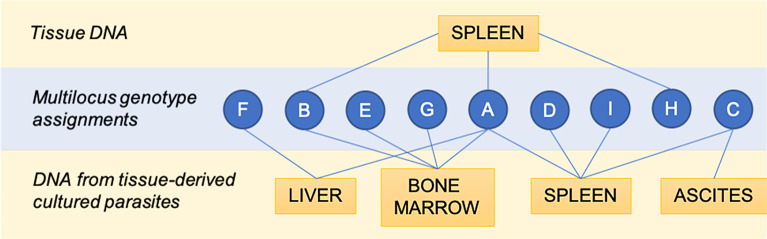
Multilocus genotypes (MLGs) assigned by MLMT found in *Leishmania infantum*-infected dogs in an endemic area of visceral leishmaniasis in Brazil. Microsatellite marker (MLMT) analysis was performed using DNA from tissue-derived *L*. *infantum* cultures or tissue DNA spleens. Due to missing data, 64 analyzed samples could not be genotyped. The remaining 22 samples were successfully assigned to an MLG (A to I). Three MLGs were found in more than one tissue. One genotype (A) was detected in all samples except those derived from ascites.

This study was conducted in a highly endemic region of VL where different genotypes and genetic populations of *L*. *infantum* have been detected previously [6, 25]. Therefore, exposure to multiple independent genotype infections during the lifetime of the host is expected. Twenty-three samples (23 of n = 86) were effectively genotyped by MLMT, and nine MLGs were detected, which were referred to as MLG A–I (S1 Table). MLGs were assigned only to samples for which all the MLMT markers were successfully amplified and determined. The same MLG was detected in different tissues (Fig 2). MLG A was the most frequently detected (n = 13) and was detected in spleen (tissue) and in the derived isolated promastigotes from spleen, bone marrow and liver. MLG B was detected in spleen (tissue) and in bone marrow-derived cultures. MLG C was detected in the derived cultures of spleen and ascites aspirate. Therefore, MLGs A, B and C were detected in different tissue types in 13, 2 and 2 samples, respectively. Other MLGs were exclusively detected in one tissue type but represented by only one sample (S1 Table). Thus, specific MLG-tissue tropism cannot be assumed.

**Fig 3:**
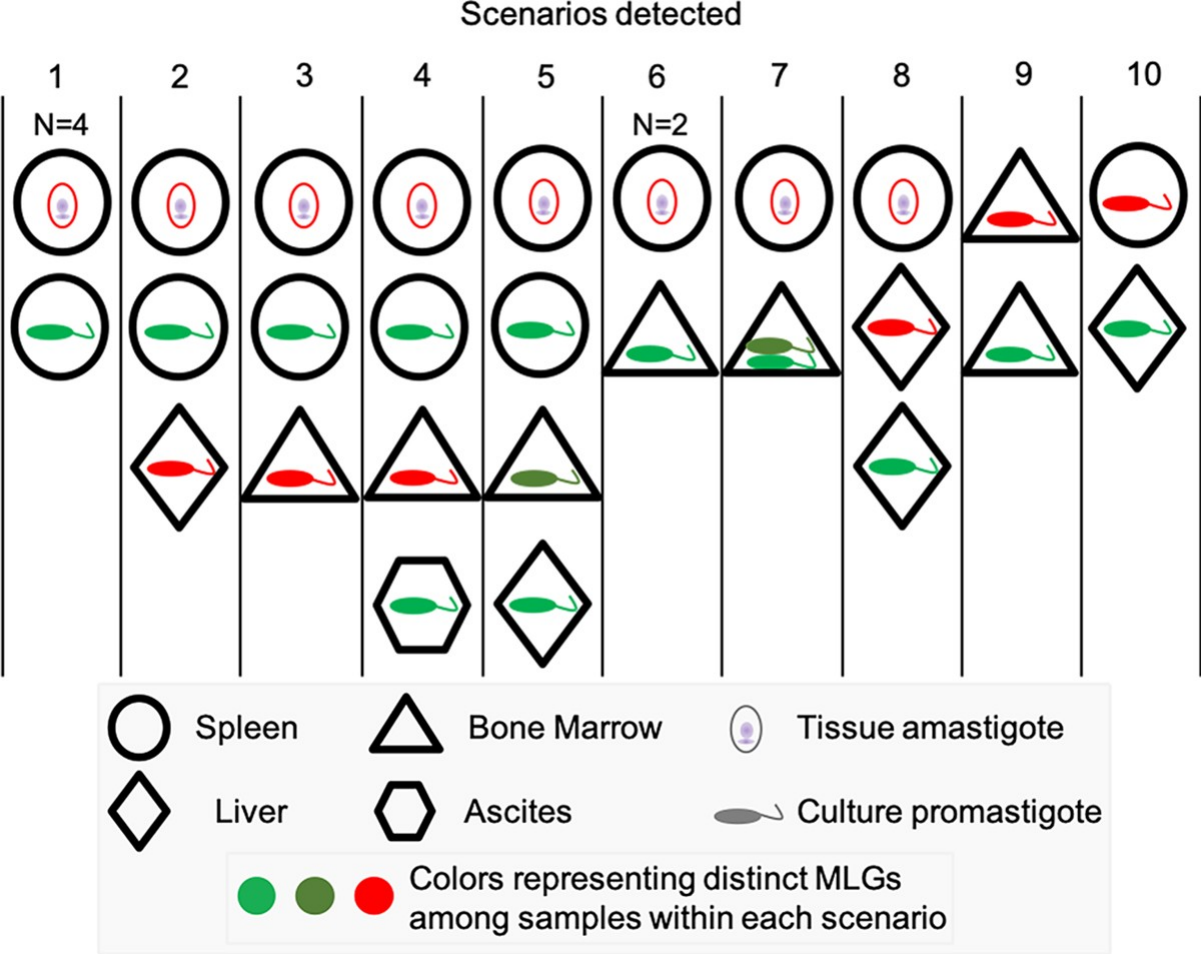
Multiple genotype *Leishmania infantum* infections detected by MLMT in paired samples from dogs from an endemic area of visceral leishmaniasis in Brazil. Microsatellite analyses were performed for 14 markers using DNA from tissue derived from cultured parasites or using tissue (spleen) DNA extracted directly from naturally infected animals. Each column represents one scenario observed among the analyzed paired samples. For example, Scenario 1 was observed in 4 dogs, in which the paired samples (spleen tissue and spleen-derived isolated promastigotes) exhibit distinct multilocus genotypes (MLGs). Scenario 2 reproduces Scenario 1 with the additional observation of the same MLG in spleen tissue and liver-derived cultures. Scenario 7 depicts distinct MLGs in spleen (tissue) and bone marrow-derived cultures, in which two distinct MLGs were codetected.

Four MLGs (A, B, E and G) were observed in bone marrow-derived *Leishmania* DNA, and two MLGs (A and F) were observed in the liver. Six MLGs were detected among the spleen samples, four of which (A, C, D and I) were found in spleen-derived cultures and three of which (A, B, and H) were identified directly from spleen tissue DNA. The results indicate that multiple genotype infections occur within a host and even within a single tissue sample (Fig 3).

In the paired sample analysis, distinct MLMT alleles were detected in 14 dogs (Table 1 and S1 Table), reflecting 10 different scenarios (Fig 3) in which infection by distinct MLGs occurred. The observations within our sample revealed i) different MLGs in two cultures derived from the same tissue (Animals 113, 116 and 121—Table 1 and Scenarios 8 and 9—Fig 3); ii) different MLGs coinfecting the same tissue and the same MLGs infecting different tissues (Animals 244, 252 and 256—Table 1; Scenarios 2, 3, 4, 5 and 7—Fig 3). It is unlikely that the *in vitro* conditions favored a specific genotype, as we also observed paired sample genotype matching. Thus, the effect of culture conditions may reflect the random filtering of parasite cell populations.

**Table 1:** Animals displaying different microsatellite alleles representing *Leishmania infantum* parasites infecting distinct tissues. For DNA isolation directly from tissues, only spleen samples were employed, but DNA was also isolated from cultured parasites isolated from diverse tissues from naturally infected dogs living in an endemic area of visceral leishmaniasis in Brazil.

Animal ID	Sample type^1^	Sample ID	Tissue^2^	Marker		Scenario (depicted in Fig 3)
				Allele 1	Allele 2	
**107**				**Lm4TA**		
	Tissue	107	Spleen	81	83	
	Strain	3130	Bone Marrow	79	83	6
**110**				**Lm4TA**		
	Tissue	110	Spleen	83	83	6
	Strain	3131	Bone Marrow	81	81	
113*				**Lm2TG**		
	Tissue	113	Spleen	140	140	7
	Strain	3132	Bone Marrow	142	142	
	Strain	3158	Bone Marrow	142	142	
				**Lm4TA**		
	Tissue	113	Spleen	-	-	
	Strain	3132	Bone Marrow	81	81	
	Strain	3158	Bone Marrow	83	83	
**116**				**Li41–56**		
	Strain	3133 – 1^st^	Bone Marrow	88	90	9
	Strain	3133 – 2^nd^	Bone Marrow	90	90	
**121**				**Li22–35**		
	Tissue	121	Spleen	94	94	8
	Strain	3134	Liver	94	94	
	Strain	3135	Liver	96	96	
				**Lm4TA**		
	Tissue	121	Spleen	83	83	
	Strain	3134	Liver	83	83	
	Strain	3135	Liver	79	79	
				**Li 71–33**		
	Tissue	121	Spleen	105	105	
	Strain	3134	Liver	105	105	
	Strain	3135	Liver	106	106	
				**Li 71–52**		
	Tissue	121	Spleen	-	-	
	Strain	3134	Liver	110	110	
	Strain	3135	Liver	108	108	
				**Li71–70**		
	Tissue	121	Spleen	100	100	
	Strain	3134	Liver	100	100	
	Strain	3135	Liver	98	98	
**124**				**Lm2TG**		
	Tissue	124	Spleen	138	142	1
	Strain	3137	Spleen	136	142	
**239**				**Li22–35**		
	Tissue	239	Spleen	94	94	1
	Strain	3201	Spleen	94	96	
**244**				**Li 71–33**		
	Tissue	244	Spleen	105	107	
	Strain	3206	Spleen	107	107	4
	Strain	3205	Bone Marrow	105	107	
	Strain	3207	Ascites	107	107	
				**Li22–35**		
	Tissue	244	Spleen	94	94	
	Strain	3206	Spleen	94	96	
	Strain	3205	Bone Marrow	94	96	
	Strain	3207	Ascites	94	96	
**251**				**Li22–35**		
	Strain	3210	Spleen	94	96	10
	Strain	3211	Liver	94	94	
**252**				**Lm4TA**		
	Tissue	252	Spleen	79	83	
	Strain	3213	Spleen	83	83	3
	Strain	3212	Bone Marrow	79	83	
**254**				**Lm2TG**		
	Tissue	254	Spleen	142	142	1
	Strain	3215	Spleen	128	142	
256*				**Lm2TG**		
	Tissue	256	Spleen	142	142	2
	Strain	3217	Spleen	144	144	
	Strain	3218	Liver	142	142	
				**Lm4TA**		
	Tissue	256	Spleen	83	83	
	Strain	3217	Spleen	81	81	
	Strain	3218	Liver	83	83	
**257**				**Lm2TG**		
	Tissue	257	Spleen	142	142	
	Strain	3220	Spleen	142	144	5
	Strain	3219	Bone Marrow	142	146	
	Strain	3221	Liver	142	144	
291*				**Lm2TG**		
	Tissue	291	Spleen	142	142	1
	Strain	3323	Spleen	142	144	
				**Lm4TA**		
	Tissue	291	Spleen	83	83	
	Strain	3323	Spleen	85	85	

^1^Sample type—type of sample employed for DNA extraction; Tissue indicates that DNA was extracted directly from a spleen fragment and Strain indicates that DNA was extracted from cultured parasites.

^2^Tissue–the tissue from which DNA was extracted or the parasite (strain) from which DNA was isolated; *Different population assignment was made after STRUCTURE analysis

As examples: i) the same MLG occurred in the spleen tissue and bone marrow-derived promastigotes (Animal 252 and Scenarios 3 and 4 in Fig 3) or liver (see Animal 256 and Scenarios 2 in Fig 3); however, the spleen-derived isolate differed. ii) Two isolates from different liver fragments of the same animal were typed as distinct MLG, and one of them was identical to the MLG from the spleen tissue (see Animal 121).

The differences in paired samples were found either in one (see Animals 124, 239, 252, 254 and 257) or two loci (Animals 244, 256 and 291). The difference in only one marker may be due to the selection of a less frequent, genetically diverse clone from the initial population. Errors cannot be ruled out, although microsatellite marker stability has already been demonstrated [26]. All mismatches in the same dog genotype were triple-checked via independent PCR and fragment analyses.

**Fig 4:**
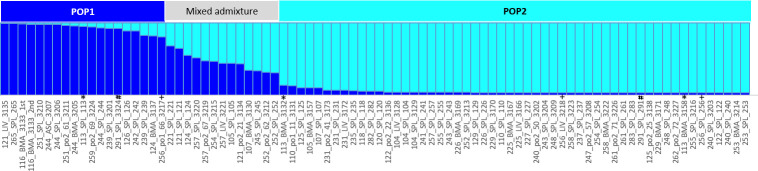
Population structure of *Leishmania infantum* infecting dogs in an endemic area of Visceral Leishmaniasis in Brazil. Data were obtained on the STRUCTURE software and processed for visualization (see *Material and Methods* section for details). MLMT was performed in 86 samples for 14 microsatellite markers using DNA extracted from *Leishmania* isolated from tissues or using DNA extracted directly from the spleen of infected dogs. Two genetic clusters were identified corresponding to POP1 (dark blue horizontal bar) and POP2 (light blue horizontal bar). Each vertical line represents one sample and is divided into the estimated proportion (length of the colour) of membership in that population. 13 samples showed admixture and we considered as POP1 or POP2 when presenting Q≥0.70 (k = 2; Fst = 0.405753; p = 0.0001). Symbols +, # and * indicate dogs 256, 291 and 113, respectively.

MLMT analysis allocated the paired samples of three dogs in different populations (Table 1 and Fig 4): Samples from Animal 113 were typed as POP1 in spleen and POP2 in the bone marrow; Animal 256 was typed as POP1 in the spleen and POP2 in the liver-derived sample; Distinct spleen samples from Animal 291 were genotyped as distinct MLMT populations.

Distinct promastigote cultures derived from the same tissue may present similar *L*. *infantum* MLG combinations, but only the most prevalent MLG is efficiently detected by MLMT. Therefore, although both MLGs originally coinfected the tissue and were successfully isolated, the culture conditions stochastically filtered the parasite cell populations, affecting the final detection of MLGs. More high-resolution approaches, such as single-cell sequencing or cloning, could be used to explore this phenomenon.

Samples analyzed herein came from dogs that mainly lived in the same municipality; thus, the low number of unique MLGs that was found is expected and suggests clonal propagation. Higher genotypic diversity was demonstrated in another study conducted in Brazil [6], but the analyzed strains were from different geographic regions and hosts. Our results are similar to those obtained by Motoie and coworkers [16] who also used MLMT to genotype *L*. *infantum* directly from dog tissue collected at different municipalities in the State of São Paulo. Of the 112 samples analyzed, Motoie et al. observed 33 MLGs (of which 20 represented unique MLGs) structured across two populations that corresponded to the Northwest and Southeast regions of the State.

The STRUCTURE analysis grouped the samples into two populations (k = 2; F_ST_ = 0.405753; p = 0.0001), which is supported by the F_ST_ value; 13 samples displayed a similar admixture for both ancestral populations. Only samples for which Q ≥ 0.70 after the CLUMPP alignments were considered to be part of POP1 or POP2 (4). In our previous study, at least two *L*. *infantum* populations were detected in Mato Grosso [6], which is the same region in which the present study was conducted. Due to missing data, comparisons with the samples analyzed in our previous study could not be performed. Even though this corroborated the results of our previous study, a higher proportion of POP2 samples (54 samples) was also observed. Conversely, POP1 comprised only 21 samples (see [6]). Based on the analysis of MLGs, there was no indication of tissue tropism in any of the populations.

In many parasitic diseases, acquired immunity due to primary infection does not always fully protect against secondary infection [27, 28]. Thus, the dogs could have been subject to multiple infections during their lifetime, as they had lived in an endemic region. Multiple genotype infections may also occur in sand flies ultimately transmitted to vertebrate hosts; however, *Leishmania* detection in sand flies is not an easy task, and methods such as MLMT are not commonly employed. It is known that sand flies can present natural mixed infections by distinct *Leishmania* species [29, 30], and experimental infections using *L*. *major* and *L*. *turanica* showed no sign of competition [31]. Recently, it was hypothesized that the particular composition of the sand fly fauna in one region in Italy may play a role in the selection of a particular *L*. *infantum* population that circulates in humans and vectors but not in dogs [32]. Although the methodology employed was different, and the results cannot be directly compared, studies conducted in Brazil indicated that the same *L*. *infantum* strain infected dogs and humans [33, 34].

In endemic areas in which different *Leishmania* genotypes are transmitted, the disease can be the result of a heterogeneous infective inoculum, which is likely the result of an accumulation of multiple independent infections [35]. Superinfections, particularly by strains with different genotypes, have been shown to be important in the pathogenesis of some parasitic diseases [27, 36]. In malaria, parasite genetic diversity and multiplicity of infection affect clinical outcomes, responses to drug treatment and naturally acquired or vaccine-induced immunity [37]. The spleen parasite loads and clinical scores of the dogs examined in the present study had been previously determined [21], but no difference in clinical signs or parasite load was observed between multiple MLG infection and single MLG infection groups. Of the 29 dogs from which paired samples were obtained, 15 presented the same MLG (the single MLG infection group). Of those, eight had high clinical scores, five had medium scores and two had low scores. Similarly, of the 14 dogs that had multiple MLG infections, six had high clinical scores, another six had medium scores and two had low scores. In both groups, most animals had high parasite loads (a parameter associated with modifications in the structure of splenic lymphoid microarchitecture and deficiencies in cytokine expression) [21].

Dogs in high endemic CVL areas are prone to multiple infections, i.e., may be coinfected by multiple *L*. *infantum* genotypes. However, such coinfections are not easily detected due to the low resolution in the technical approaches available. This could lead to the underestimation of the frequency and diversity of multiple genotype infections. Studies on malaria patients have suggested that the multiplicity of infection usually reflects the degree of transmission intensity, although the association is not linear [38, 39]. In endemic regions for malaria, mixed infections represent more than 70% of infections [14]. In a population infected by *Plasmodium* species, multiclonal infections were detected in 62.2% of the samples by long-fragment deep sequencing and 45.9% of the samples by short-fragment deep sequencing, but only 5.2% of the samples were successfully identified by microsatellite markers [37]. The detection of different MLMT profiles in parasites collected from human patients who have presented more than one episode of VL might reflect a clinical relapse or reinfection [40]; also, multiclonal infections cannot be ruled out. To date, very few studies have considered the occurrence of mixed infections in *Leishmania*. Recently, chromosome copy number variation was demonstrated among clones isolated from an HIV-VL patient. Variation was also observed in the k26-PCR amplicon, suggesting mixed infection. In addition, different karyotypes were observed in strains isolated from different organs (i.e., the spleen, skin and bone marrow) from the same patient [41]. Differences in the chromosome ploidy of strains isolated from different organs may be the result of clonal selection of parasites in the host due to specific selective pressures at the infection site. Genetically diverse infections may be common in many VL endemic areas; however, it is important to address the effects that coinfected strains have on each other in a natural host-parasite system.

The present descriptive study found evidence of multiple MLG natural infections by *L*. *infantum* in hosts living in a high-transmission area for VL. Further investigations on the effects of multiple infections are needed. Studies on other pathogens have shown that coinfections affect pathogen transmission and virulence, thus influencing disease dynamics [42]. Multiple genotype infections may have an impact on host immune responses [8, 43] and the effectiveness of disease control [44]. Notably, as observed in the antimony deposition in different organs of glucantime-treated rhesus monkeys, the pharmacokinetics of leishmanicidal drugs may vary depending on the organ [45]. This could lead to the development of resistance or selection if a more resistant genotype is found in an organ showing reduced levels of drug accumulation.

The results presented herein confirm the hypothesis raised in this study by showing the presence of multiple infections (multilocus genotypes, as inferred by microsatellite analysis) in dogs infected by *L*. *infantum* living in a highly endemic area for VL, which may contribute to the evolution of virulence in naturally infected dogs. Additionally, differences between clinical samples and derived culture isolates were also described. However, the understanding of the physiological and evolutionary consequences of these infections remains limited.

## Supporting information

S1 Table Sample information and data for the molecular analysis performed by using multilocus microsatellite typing (MLMT). The genotypic composition of *Leishmania infantum* in naturally infected dogs was evaluated in parasites obtained from different tissues with or without *in vitro* expansion. The reference size in length of each allele of the microsatellite markers is presented. The observed length is presented for each animal.
(XLSX)

S2 Table Detailed information for the 14 microsatellite markers used. (DOCX)
