# Supplementary material for: Occurrence of multiple genotype infection caused by Leishmania infantum in naturally infected dogs
Source: PLoS Negl Trop Dis. 2020 Jul 27;14(7):e0007986. doi: 10.1371/journal.pntd.0007986 (PMC7410330; doi:10.1371/journal.pntd.0007986)
Supplement: S2 Table — (DOCX) [file pntd.0007986.s002.docx]

| **Marker** | **(5'-3')** | **(5’-3’)** | **AT** | **Repeat array** | **Fragment size (pb)** |
| --- | --- | --- | --- | --- | --- |
|  |  |  |  |  |  |
| Li22-35 | CTTGATGTTCGGGTTAGCAAGT | ATGCACACCAAAAATCATGTG | 52 | CA | 92/100 |
| Li23-41 | GATCGGAGGTGACAGCGT | CCTTTAACTGCCAGTGCG | 52 | GT | 77/87 |
| Li41-56 | TTGCTTCATGATAACAACTTGG | CCTGTTGGTGTGAGTTCGTG | 50 | CA | 90/92 |
| Li45-24 | GCGCCTACAGGCATAAAGGA | CTGGCGCATCAACGGTGT | 54 | CA | 81/107 |
| Li46-67 | TCTTCTTTCGTTAGCTGAGTGC | CTGTATCACCCATGAGGGGC | 50 | CA | 80/82 |
| Li71-5/2 | GCACGGTCGGCATTTGTA | GATAAACGAGATGGCCGC | 56 | CA | 110/110 |
| Li71-7 | GCTGCAGCAGATGAGAAGG | GTGAGAAGGCAGGGATTCAA | 50 | CA | 92/102 |
| Li71-33 | CTCCTTTCACACCGCCTCT | GAGAGAAGACGAGCCGAAGT | 50 | TG | 103/107 |
| Lm2TG | AAAAAGCGAGGAATGAAAGAA | TCCCTCCCCTCTACAACCTT | 53 | TG | 138/152 |
| Lm4TA | TTTGCCACACACATACACTTAG | GTAGACGACATCGCGAGCAC | 54 | TA | 77/83 |
| TubCA | GGCGTGGTTGCTAAACTGAT | GCCTGCGCACACAGAGAC | 58 | CA | 80/82 |
| CS20 | CGTTGGCTGTTGATTGTGTA | GCGTGGCAATCTCCTCATT | 56 | TG | 83/83 |
| LIST7031 | GCGGGAGTCGTCTCTCTGTT | AACGTGCAGTACGCAAGGAC | 58 | CA | 111/111 |
| LIST7039 | CACTCTTTCGCTCTTTGCTG | TGGCTCCACAATATCGACAA | 58 | CA | 205/211 |

AT, annealing temperature; bp, base pairs
